# Pasireotide and octreotide in the treatment of severe late dumping syndrome

**DOI:** 10.1002/ccr3.1025

**Published:** 2017-08-22

**Authors:** Alireza Mohammadi, Raashda A. Sulaiman, Ashley B. Grossman

**Affiliations:** ^1^ Oxford Centre for Diabetes, Endocrinology and Metabolism Churchill Hospital University of Oxford Oxford UK; ^2^ Department of Clinical Biochemistry Queen Elizabeth Hospital Birmingham UK

**Keywords:** Dumping, hypoglycaemia, octreotide, oesophagectomy, pasireotide

## Abstract

Hypoglycemia due to late dumping is a significant problem postoesophagectomy but may not always be diagnosed sufficiently early. It can be difficult to treat and may severely compromise quality of life. The combination of diazoxide and octreotide or more probably pasireotide may transform the patient's life and should be considered in all problematic cases.

## Introduction

The dumping syndrome refers to symptoms and signs that occur when food reaches the small bowel too rapidly: The condition most commonly occurs after partial or total gastrectomy or esophageal surgery 1. With the increase in bariatric surgery, it is to be anticipated that more dumping syndromes will be seen.

The symptoms of dumping syndrome can be classed as early or late, depending on how soon after ingestion they occur. The early symptoms, usually within 30 min of eating, comprise both gastrointestinal (including abdominal pain, diarrhea, borborygmi, nausea, and bloating) and vasomotor symptoms. It is generally thought that these relate to the excessively rapid entry of food into the gastrointestinal system and the inability of the gut to process them as rapidly as necessary.

Late dumping symptoms include perspiration, palpitations, hunger, fatigue, confusion, aggression, tremor, and syncope. They occur 2–3 h after ingestion of a meal and have been attributed to reactive hypoglycemia.

Late dumping as a cause of hypoglycemia has been increasingly reported, and several patients have been reported to suffer from hyperinsulinemic hypoglycemia with nesidioblastosis after gastric bypass surgery; these patients were not responding to conventional treatment for the dumping syndrome 2.

Dietary measures are the first step in managing the dumping syndrome. Medical treatment includes diazoxide, acarbose, and the use of the somatostatin analog, octreotide. However, there remain a group of patients who remain recalcitrant to all such therapies.

Pasireotide is a multireceptor targeting somatostatin analog (SSA) that binds with high affinity to four of the five somatostatin receptor subtypes. A major adverse event is hyperglycemia and increased HbA_1C_, principally by reducing the secretion of both glucagon‐like peptide (GLP)‐1 and glucose‐dependent insulinotropic polypeptide (GIP), as well as insulin directly 3, 4. Recently, a small pilot study showed that pasireotide administration in patients with the dumping syndrome increased nadir glycaemia and peak glycaemia compared with placebo 5.

We report a patient who developed severe hypoglycemic episodes post‐oesophagectomy who failed to respond to conventional measures, but is now under treatment with pasireotide with moderately effective hypoglycemic control.

## Case

A 57‐year‐old lady underwent minimally invasive oesophagectomy in 2009 for a moderately differentiated squamous cell carcinoma of the esophagus. She had no other significant medical history and was not on any medications. She was an ex‐smoker with past history of excessive alcohol intake. Two months following surgery her family noted that she became confused 1–2 h after meals. She had several attendances at the Emergency Department with collapses over a 2‐year period: She reported feeling unwell, dizzy, and sweaty and disoriented, with headache before each collapse. She also experienced bloating and nonspecific abdominal pain during the episodes. Her symptoms started 30 min to 2 h after meals and disappeared after eating confectionery.

On one occasion, she was reported to be hypoglycemic during one of these attacks by paramedics in 2010. She was subsequently seen in the gastroenterology clinic for suspected dumping syndrome. However, there was no improvement in her symptoms following dietary advice. She was referred to the metabolic clinic for a trial of uncooked corn starch to ameliorate her symptoms, and this did not work either. In response to a standard meal test, she showed marked hypoglycemia with inappropriately raised insulin levels. Her fasting glucose of 4.2 mmol/L fell to 1.6 after 90 min and 1.5 after 150 min. Plasma insulin was raised at 102 pmol/L and C peptide at 841 pmol/L after 150 min. In spite of significant hypoglycemia during the test she remained conscious, although her husband noticed mild confusion in her conversation. Sulfonylurea and insulin antibody screen were negative. Computerized tomogram (CT) scanning of the abdomen, endoscopic ultrasound of the pancreas, and ^18^FDG‐positron emission tomography (PET) scanning were all normal. She was started on diazoxide with no improvement in her symptoms. At that stage she was referred to Oxford for selective pancreatic artery calcium stimulation testing.

She was admitted to this hospital in February 2012. She showed persisting hypoglycemic episodes after meals. Her vital signs, physical examination and routine hematological and biochemical testing including thyroid function tests and cortisol were normal. Magnetic resonance imaging (MRI) of the abdomen with gadolinium showed a subtle questionable rounded area of abnormal enhancement lying anteriorly at the junction of the body and head of the pancreas. There was no clear step‐up in blood insulin levels during selective pancreatic artery calcium stimulation test, which essentially ruled out the possibility of insulinoma or noninsulinoma pancreatogenous hypoglycemia syndrome (NIPHS). It was clear that we were dealing with a postoesophagectomy dumping syndrome. Since she had had diazoxide 50 mg tds for 5 months without any improvement in her symptoms or in capillary blood glucose (CBG) readings, acarbose was prescribed in an attempt to delay carbohydrate absorption, but 70% of her 2‐h postdinner CBG readings were still less than 4 mmol/L. She then was started on subcutaneous octreotide 100 *μ*g tds injections; these reduced her hypoglycemic episodes to 24% of two‐hour postdinner CBG readings. Short‐acting octreotide was replaced with monthly lanreotide Autogel 90 mg after 10 weeks. Immediately after the first lanreotide injection her hypoglycemic episodes *increased*, so we added regular octreotide 100 *μ*g tds which she took for 16 days, and then discontinued the Lanreotide Autogel. After a further 18 days, her octreotide was increased to 200 *μ*g tds, and then diazoxide was added and titrated up to 100 mg tds. The combination of diazoxide 100 mg tds and octreotide 200 *μ*g tds reduced her hypoglycemic episodes to 10% of her 2‐h postdinner CBG readings.

A trial of pasireotide was planned. Pasireotide alone at a dose of 900 *μ*g bd was poorly effective, with 79% of two‐hour postdinner readings showing hypoglycemia. The best result was the combination of pasireotide 900 *μ*g bd plus diazoxide 100 mg tds, which reduced the percentage of hypoglycemic episodes to 8% and increased her average CBG reading to 8.06 mmol/L and maximum CBG reading to 24.1 mmol/L. She remains well controlled on this combination in terms of the avoidance of hypoglycemia some 4 years after referral (Table 1).

**Table 1:** Two hour post dinner capillary glucose reading on various treatment regimens

Treatment	Day	No of 2‐h postdinner CBG readings	No of 2‐h postdinner hypos	Percentage of hypos/2‐h postdinner readings	Minimum CBG	Maximum CBG	Average CBG
No Medication	9	8	6	75	2.2	4.7	3.4
Acarbose 100 mg/TDS	119	102	71	69.6	1.2	9.3	3.47
Octreotide 100 *μ*g/TDS	85	66	16	24.24	1.8	16.2	5.83
Octreotide 200 *μ*g/TDS	18	12	6	50	2.1	8.3	4.57
Lanreotide 90 mg monthly	39	37	19	51.35	1.6	12.6	4.55
Pasireotide 600 *μ*g/BD	28	24	19	79.16	1.6	8.7	3.49
Pasireotide 900 *μ*g/BD	14	14	11	78.57	1.9	5.2	5.69
Octreotide 200 *μ*g/TDS+ Diazoxide 50 mg/TDS	41	34	13	38.23	2.3	11.9	5.11
Octreotide 200 *μ*g/TDS + Diazoxide 100 mg/TDS	173	130	13	10.0	1.3	14.8	6.75
Octreotide 100 *μ*g/TDS + Lanreotide 90 mg	16	15	8	53.33	2.3	9.7	4.24
Pasireotide 900 *μ*g/BD + Diazoxide 50 mg/BD	64	46	1	36.95	2.9	9.3	4.65
Pasireotide 900 *μ*g/BD + Diazoxide 100 mg/TDS	195	143	12	8.39	1.8	24.1	8.06

## Discussion

Hypoglycemia is a significant problem postoesophagectomy but may not always be diagnosed sufficiently early. Our patient had endogenous hyperinsulinemic hypoglycemia. The main differential diagnoses were insulinoma, noninsulinoma pancreatogenous hypoglycemia syndrome (NIPHS) or the presence of antibody to insulin or insulin receptor. She did not have fasting hypoglycemia, which is usually seen in insulinoma. CT scanning of her abdomen with contrast was unremarkable. Endoscopic ultrasound scan of the pancreas and FDG‐PET scanning showed no evidence of insulinoma. In view of the postprandial hypoglycemia, and initial negative imaging, NIPHS was considered as a possible cause of her symptoms. NIPHS is known to develop after bariatric surgery and oesophagectomy. Rapid passage of food to ileum results in increased levels of GLP1 which may cause proliferation of islets and β‐cell hyperplasia. However, subsequent MRI of her pancreas showed a questionable area in pancreas raising the suspicion of an insulinoma. Nonetheless, a lack of a calcium‐stimulated insulin gradient ruled out both insulinoma and NIPHS.

We tried acarbose and diazoxide, but neither alone was clinically useful. Both octreotide and pasireotide were also ineffective as sole agents. In our patient, octreotide or pasireotide in combination with diazoxide was the most effective agents, with a suggestion that pasireotide may be preferable. She preferred the twice‐daily regimen of pasireotide, which has the advantage of not requiring refrigerator storage. We have to date followed her up for nearly 4 years, and she remains in good health with currently only very occasional hypoglycemic episodes and a good quality of life. She has not experienced any adverse events with the pasireotide, and she remains recurrence free from her cancer. She has no gastrointestinal symptoms, and her liver function tests remain normal.

In summary, the late‐dumping syndrome can be difficult to treat and may severely compromise quality of life postoesophagectomy. The combination of diazoxide and octreotide and particularly pasireotide my transform the patient's life and should be considered in all problematic cases.

## Authorship

AM: looked after the patient in Tertiary centre and in clinic, collected data, did literature search, and wrote the manuscript. RAS: managed the patient locally, provided patient's data, and revised the manuscript. ABG: looked after the patient in tertiary centre and in clinics, had the idea of using Pasireotide, authorized its use, and revised the manuscript.

## Conflict of Interest

None declared.
